# Wellness Warriors: a qualitative exploration of healthcare staff learning to support their colleagues in the aftermath of the Australian bushfires

**DOI:** 10.1080/17482631.2023.2167298

**Published:** 2023-01-19

**Authors:** Andrea Knezevic, Katarzyna Olcoń, Louisa Smith, Julaine Allan, Padmini Pai

**Affiliations:** a School of Health and Society, University of Wollongong, New South Wales, Australia; b Deakin University; c Rural Health Research Institute, Charles Sturt University, Orange, New South Wales, Australia; d Illawarra Shoalhaven Local Health District

**Keywords:** Bushfires, post disaster, healthcare providers, occupational trauma, burnout, workplace wellness, mental health and wellbeing

## Abstract

**Purpose:**

Healthcare staff are on the frontline during disasters despite any personal adversity and vicarious trauma they may be experiencing. *Wellness Warrior* training is a post-disaster intervention developed in response to the 2019-2020 Australian bushfires to support staff in a rural hospital located on the South Coast of New South Wales, Australia.

**Method:**

This study explored the experiences and perspectives of 18 healthcare staff who were trained to provide emotional and peer support to their colleagues in the aftermath of a crisis. All the *Wellness Warriors* participated in semi-structured interviews between March and April 2020. Data were analysed using the reflexive thematic approach.

**Results:**

Healthcare staff reported developing interpersonal skills around deep listening and connecting with others which allowed for hearing the core of their colleagues’ concerns. The training also helped staff to feel differently about work and restored their faith in healthcare leadership.

**Conclusion:**

*Wellness Warrior* training provided staff with knowledge and skills to support their colleagues in the aftermath of a natural disaster and later during the COVID-19 pandemic. As such, these findings suggest that peer support programs such as *Wellness Warriors* could be one way healthcare organisations can attempt to alleviate the psychological impact of natural disasters.

## Introduction

Healthcare staff are on the frontline during times of disaster such as bushfires and pandemics. Despite any personal adversity and/or vicarious trauma, they are likely to experience during and after disasters (Beck, 2011; Brooks et al., 2019; Crabbe et al., 2004; Cronin et al., 2007), healthcare workers are required to continue supporting their patients in their recovery. In early 2020, there was little evidence of workplace interventions, particularly in hospitals, striving to ameliorate the immediate emotional effects and ongoing needs and concerns of healthcare staff resulting from working during disasters (Du Plooy et al., 2014; Yuma et al., 2019). However, the implementation of hospital interventions seeking to support frontline staff has been increasing in the face of traumatic events other than natural disasters, including the COVID-19 pandemic (Pollock et al., 2020).

The purpose of this paper is to explore the experiences of healthcare staff at a small rural hospital located in the Illawarra Shoalhaven Local Health District (ISLHD), New South Wales (NSW) who took on the role of *Wellness Warriors* to support the wellbeing of their work community in the aftermath of the 2019–2020 Australian bushfires. A total of 499,621 ha of land were lost as a result of the fires, with 312 homes destroyed and 173 damaged in the surrounding suburbs of the small town (SBS News, 2020; Shoalhaven City Council, 2020). Three of the homes that were destroyed belonged to hospital staff. A state coronial inquiry confirmed that three people died as a result of the fires (ABC News, 2021). The heavy rains, which extinguished the fires after 74 days, resulted in another disaster—flooding (Shoalhaven City Council, 2020). The impact of the two disasters on the rural community and surrounding areas was unprecedented.

Financial and human resources were immediately mobilized within the local health district to respond to the psychological impact of the bushfires and floods on the hospital staff. As a result, a wellness programme named SEED was co-designed with the staff as a whole-hospital approach to support collective healing, wellbeing and resilience (Pai et al., 2022). The SEED Program is “a workplace wellness model that strives for staff to experience more meaning, happiness and connectedness at work” (Pai et al., 2022, p. 3). The development of SEED was guided by a strengths-based approach (Healy, 2014) and post-traumatic growth (PTG) theory (Tedeschi & Calhoun, 1995). SEED aimed to facilitate growth for staff after trauma including “positive changes to a sense of personal strength, appreciation of life, spiritual change, new possibilities, and relating to others” (Harms et al., 2018, p. 418). The *Wellness Warrior* training was one of five SEED initiatives implemented to provide immediate support to staff following the bushfires. Eighteen healthcare staff from the hospital took part in a two-day *Wellness Warrior* training to gain skills in strengths-based ways of creating a supportive work environment in the aftermath of crisis. *Wellness Warriors* were selected either through expression of interest to participate in the training or being recommended by the Director of Nursing and Midwifery Services/Operations Manager, their Nurse Unit Manager, Clinical Nurse Educators, or work colleagues. However, participation in the training was voluntary. This paper describes their experiences and perspectives of *Wellness Warriors* who learned how to support their colleagues in times of disaster.

### Literature review

Healthcare staff serve an essential role in providing care during and in the aftermath of critical events, including natural disasters (Scrymgeour et al., 2016). Healthcare staff in regional and rural areas often live and work in the same community and are experiencing the disaster themselves—experiencing the same physical and psychological reactions, fear and uncertainty as those who they are caring for (Richardson & Ardagh, 2013). Working in conditions of extreme stress during and in the aftermath of natural disasters, healthcare staff experience personal losses, injuries and other stressors including vicarious trauma brought about by the disaster (Beck, 2011; Benedek et al., 2007). Studies conducted in the aftermath of disasters, such as the 2010 New Zealand earthquake, or the 2005 Hurricane Katrina in the US, showed that healthcare staff found themselves in the role of both practitioner and victim (Danna et al., 2010); feeling overwhelmed, and losing a sense of safety (Richardson & Ardagh, 2013). During disasters, healthcare staff are not only managing their own immediate sense of personal loss but also need to take on new roles required of them during these times, including psychosocial support to others, leadership, and inter-professional collaboration (Dietchman, 2013; Filmer & Ranse, 2013; Waugh & Streib, 2006).

Some strategies to address compassion fatigue, burnout and vicarious trauma post-disaster have been trialled and collective action has been identified as important (Umeda et al., 2020; Yuma et al., 2019). Community interactions and connections are a significant “predictor of successful coping in the wake of a disaster” (Wlodarczyk et al., 2017, p. 3). To implement post-disaster interventions in a healthcare setting, team-based strategies can promote a supportive workplace atmosphere and foster situated resilience (Brooks et al., 2019; Wiig et al., 2019). Situated resilience develops on the operational frontline after a disruptive event and involves “mobilizing and combing existing sociotechnical resources (e.g., knowledge and skills)” to recover from the event (Wiig et al., 2019, p. 17). Collective strategies such as implementing a peer support system, facilitating interactive group discussions or wellness activities are considered promising psychosocial approaches to building a resilient team (Umeda et al., 2020; Yuma et al., 2019). Furthermore, employing team-building strategies post-disaster can support healthcare staff in coping with extreme and traumatic stress, burnout, and fatigue in future disasters (Brooks et al., 2019; Umeda et al., 2020; Yuma et al., 2019). These approaches to situated resilience were effective in reducing distress in healthcare staff following Hurricane Sandy in 2012 and Harvey and Maria in 2017 (Wlodarczyk et al., 2017; Yuma et al., 2019). However, there is limited literature exploring the operationalization of such interventions in other contexts.

While collective strengths-based wellness interventions are indicated to support the wellbeing of healthcare staff immediately after natural disasters (Umeda et al., 2020; Yuma et al., 2019), there are no studies from Australia, where natural disasters are common and recurring, exploring such approaches. Very little is known about the effect of workplace wellness interventions on healthcare workers as there are limited studies available (Pollock et al., 2020). Therefore, this study aimed to answer the following research questions: 1) What were the experiences and perspectives of healthcare staff who participated in the *Wellness Warriors* intervention? 2) What knowledge and skills did healthcare staff gain as a result of the *Wellness Warrior* training to support workplace wellness in the aftermath of a natural disaster? And 3) What was the role of hospital leaders in promoting staff wellness in the aftermath of a natural disaster?

## Methods

This study used an exploratory-descriptive qualitative methodology, which is particularly effective for researching topics, which are under-investigated in healthcare (Hunter et al., 2019; Sandelowski, 2000). Semi-structured interviews were conducted with healthcare workers who participated in the *Wellness Warrior* training run by the local health district. All participants provided written consent for participation and the Human Research Ethics Committee of NSW Health and University of Wollongong granted ethical approval (2020/ETH00867). The research team consisted of an honours student, a university academic, and a researcher and clinician from the local health district who supervised the student’s work. An honours’ student is a university student undertaking an additional qualification to build on their undergraduate studies by completing a self-directed research project (The University of Sydney, 2022).

### Study background

The local health district covers roughly 250 km of the southern coastal strip of NSW, Australia (New South Wales Government, 2021). The district has a workforce of more than 7,300 employees across 8 hospitals and community services, providing healthcare to around 400,000 residents of the Illawarra Shoalhaven region (Illawarra Shoalhaven Local Health District, 2019). The small rural hospital where this study was conducted is an acute facility providing inpatient, outpatient and emergency services to approximately 16,865 people residing in the local community and surrounding suburbs (Australian Bureau of Statistics (ABS, 2017). There are approximately 150 staff employed at the hospital, most of whom are residents of the local community and surrounding areas (Mackay et al., 2021).

### Description of the Wellness Warrior training

The *Wellness Warrior* training was facilitated in collaboration with an experienced social worker and a senior lecturer of nursing. The training was based on the principles of the fifth strategy of The Virtues Project, the “Art of Companioning”, focusing on becoming a better listener by listening to understand and holding space for others (The Virtues Project, 2022). The overarching aim of the training was for healthcare staff to become *Wellness Warriors*, people who could provide immediate emotional and peer support to their colleagues during a time of crisis. The process for *Wellness Warriors* to approach their colleagues was informal and did not require a referral. *Wellness Warriors* would either directly approach their colleagues that were working on the same shift to check on their wellbeing or would be approached by staff that wanted emotional or peer support.

The training was facilitated as a 16-h interactive workshop over a period of 2-days. To ensure that the *Wellness Warriors* were able to immerse themselves in the 2-day training without potential interruptions on the ward, a local community centre, 5 km away of the hospital, was selected as a suitable venue for the training. The venue is managed by community members who offered to host the training to support the hospital staff. Most of the activities took place with the group siting in a circle. Some of the activities facilitated in the *Wellness Warrior* training were individual-focused and aimed at promoting self-reflection and self-awareness, whereas others involved groupwork aimed at learning and practicing listening skills and companioning. Day 1 focused on: a) taking time out from the workplace; b) identifying individual core values and strengths; c) building skills around deep listening; and d) building connections with others by acknowledging their strengths. Day 2 involved: a) a labyrinth walk to practice self-reflection skills; b) the use of humour as a coping skill; c) compassionate leadership; and d) using art to process and share stories of the bushfires. To ensure that all staff at the hospital were aware of the *Wellness Warriors*, a poster with names of the 18 *Wellness Warrior* trainees was displayed on the walls of the hospital. Management also informed all hospital staff of the *Wellness Warrior* training and the role of the *Wellness Warriors* in the workplace at daily huddles, meetings and in a whole of hospital email.

A one-day follow-up and evaluation workshop was held one-month after the initial *Wellness Warrior* training. The aim of the follow-up was to ensure that staff were supported in their role as *Wellness Warriors*, gained confidence in providing emotional and peer support and developed a deeper understanding of strengths-based ways of working. Interviews were conducted two-weeks after this follow-up session.

### Recruitment and sample characteristics

The study utilized a purposeful sampling approach (Palinkas et al., 2013) to identify and recruit staff who were trained as *Wellness Warriors* at Milton Ulladulla Hospital. The *Wellness Warrior* facilitator informed all *Wellness Warriors* of the study, and those who expressed interest were contacted by the researcher and emailed a Recruitment Letter and Participant Information Sheet (PIS). All 18 participants of the *Wellness Warriors* training consented to participate in the study.

Study participants represented a range of roles in healthcare, including a Health and Security Assistant (*n* = 1), Clinical Nurse Educators (*n* = 2), Registered Nurses (*n* = 8), an Endorsed Enrolled Nurse (*n* = 1), Enrolled Nurse (*n* = 1), Occupational Therapists (*n* = 2), Pharmacist (*n* = 1), an Administration Officer (*n* = 1), and a Nursing Manager (*n* = 1). All participants were employed by the hospital ranging from 4 months to 20 years. The majority of the participants were female (*n* = 14). Participants had resided in the community (*n* = 4) and surrounding areas (*n* = 14) for most of their lives.

### Data collection

Eighteen semi-structured interviews were conducted by the first author via telephone over a period of four-weeks (March 2020 – April 2020). Participants were permitted by the hospital management to complete the interview during work hours in a private room at the hospital to ensure confidentiality. The interview guide included open-ended questions such as: “Why did you get involved in the *Wellness Warrior* training?”, “What did you learn during the *Wellness Warrior* training?” and “What kind of ongoing support do you need to be a *Wellness Warrior*?” (For details, see the Supplemental Material) Interviews lasted between 30 and 45 minutes (average 35 min) and were audio-recorded and transcribed by the first author. To promote critical reflection following each interview, the first author wrote detailed field notes using Edward De Bono’s six modes of thinking (Khataybeh & Salem, 2015).

### Data analysis

Consistent with an exploratory-descriptive methodology, a low inference approach to data analysis was used (Sandelowski, 2000). This meant that researchers strived to retain the original meaning of data and did not apply any conceptual or theoretical frameworks to guide the analysis. The overarching approach was reflexive thematic analysis, which as explained by Braun and Clarke (2021) involves “a six-phase process for data engagement, coding and theme development” (p. 4). De-identified transcripts and field notes were uploaded into the QSR NVivo 12 software for analysis. The first author utilized the six-phase process which involved familiarization with the data by reading and re-reading the transcripts and systematically generating the codes. The first author met with authors PP and LS regularly during this process to review and discuss the codes and solve any disagreements for example, the first author initially named the first theme “Listening to Understand”. However, the authors PP and LS believed it did not capture the findings clearly and a consensus was reached to rename the theme to “Pausing to Listen”.

## Findings

**Figure 1.**
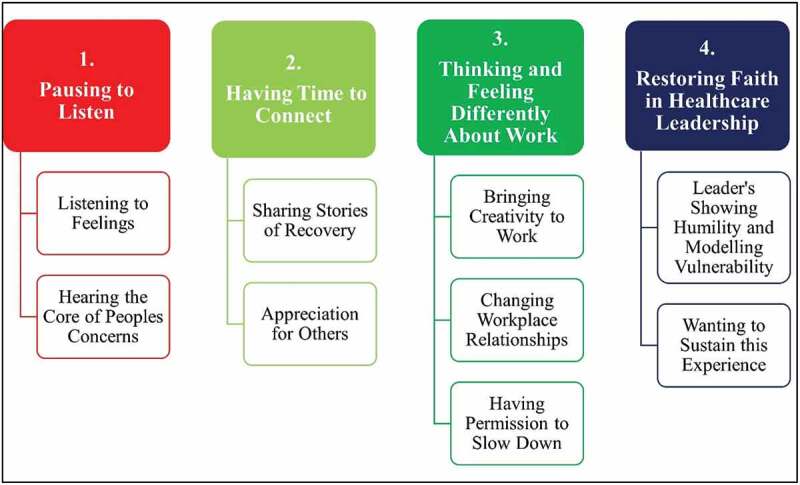
Four themes and sub-themes.

The study findings indicated that healthcare staff trained as *Wellness Warriors* developed new interpersonal skills and strategies around deep listening and connecting with others. This allowed them to think and feel differently about work and restored their faith in healthcare leadership. Four themes and nine sub-themes that were identified in the data are depicted in Figure 1 below. Research participants will be referred to as “*Wellness Warriors*” in the findings. When quoted, *Wellness Warriors* will be identified by their assigned number.

### Pausing to listen

*Wellness Warriors* described “pausing to listen” as a fundamental skill to support themselves and others while working in healthcare following a disaster. *Wellness Warriors* found that when they paused to listen to other healthcare staff, their patients, and carers they regained a sense of purpose and fulfilment in their role. *Wellness Warriors* described pausing to listen in two ways: through “listening to feelings” and “hearing the core of people’s concerns”.

#### Listening to feelings

*Wellness Warriors* defined an aspect of pausing to listen as listening to feelings. *Wellness Warriors* highlighted that listening to feelings is more than verbal communication; it is about listening to more than words—it involves observing non-verbal cues. Several *Wellness Warriors* described how the *Wellness Warrior* training provided them with strategies for identifying emotions through body language and offered a structure to talk about feelings. As one *Wellness Warrior* explained, it supported them to better relate to their colleagues: “People are a lot more open and vulnerable … it seems like a shield has lifted and we can see each other as more total human beings … I think it [*Wellness Warrior* training] has 100% improved the culture at work” (05). Several *Wellness Warriors* described the training as allowing them to reflect on their role as healthcare workers and consider new ways of interacting with others.

#### Hearing the core of people’s concerns

For many *Wellness Warriors* pausing to listen resulted in an ability to “hear the core of people’s concerns” (06) which was described as particularly useful. In their managerial roles, *Wellness Warriors* found that hearing the core of people’s concerns provided them with a structure to support their colleagues during times of fear and uncertainty in the workplace: “Just being able to try and keep that calm and listen to what their concerns are because they’re concerns are definitely valid” (04). This was further emphasized by a *Wellness Warrior* stating that “most fear as you know is of the unknown, which is where it perpetuates from, so if you’re able to let people talk it out and come to their own conclusions as well as support their feelings, they calm down” (01). Consequently, learning to hear the core of people’s concerns in the aftermath of the bushfires created more meaningful interactions between the *Wellness Warriors* and their colleagues. By “listening to feelings” and “hearing the core of people’s concerns”, the *Wellness Warrior* training enhanced *Wellness Warriors* interpersonal skills and strategies to support themselves and others in times of crisis.

### Having time to connect

*Wellness Warriors* expressed the importance of having time to connect with their colleagues in the aftermath of the bushfires to share stories of recovery and show their appreciation for one another. *Wellness Warriors* shared that they learnt the significance of “micro exchanges” which involved expressing their acknowledgement and gratitude, for example: “The small things in life that you do for somebody, thanking somebody for something they may have done or how they make you feel … ” (01). They also described that to them having time to connect was about finding meaning in interactions with their colleagues: “Saying hello to people with more meaning and more connections” (03). Many of the *Wellness Warriors* highlighted that the *Wellness Warrior* training itself created opportunities for connection between team members, by providing a safe space where they could participate in creative and fun activities. As some *Wellness Warriors* explained: “We got to know people we didn’t know before … there was so much kindness … people just showed qualities I’ve never seen in some of them before” (12). *Wellness Warriors* would regularly meet in pairs or small groups following the training, often to debrief after emotive conversations with their colleagues and/or to seek peer support from one another.

#### Sharing stories of recovery

In the aftermath of the bushfires, sharing stories of recovery was described by *Wellness Warriors* as an important aspect of having time to connect. They explained that both the physical and emotional environment of the training, such as sitting in a circle, played a crucial role in the way people shared their stories of recovery. Specifically, the first day of the *Wellness Warrior* training focused on identifying individual core values and strengths, but also acknowledging that strengths can sometimes become our weakness. One of the *Wellness Warriors* shared how this activity made her realize how being a responsible person is both, her core strength and weakness:

Because my responsibility was given to me at a young age, unbeknown to my colleagues, unbeknown to my management, why do I always put my hand up, why do I always offer, why do I jump in when there’s a need … the penny dropped … it [the session] made me understand why I put my hand up to do more hours than others during the fires, why I felt compelled when I saw a colleague struggling, I found it my responsibility… I learnt through the wellness training … that I needed balance for my mental wellbeing. This activity helped me see that which I’ve never been shown before (06).

Activities in the training such as this one supported *Wellness Warriors* to expand their self-awareness and strengthen their connections with colleagues.

#### Appreciation for others

Having time to connect also gave *Wellness Warriors* the opportunity to appreciate each other more. *Wellness Warriors* described a specific exercise during the training which involved them standing in the centre of a circle and thanking one another. Several *Wellness Warriors* reflected on this exercise as an opportunity to show appreciation and gratitude for their colleagues, which they may have not done before: “We got into a circle amongst everybody else and did a virtue acknowledgement for something we had noticed in somebody else … it was a real wake up call for all of us” (01). Another *Wellness Warrior* described how validating this activity felt: “For a lot of us it was sort of like ‘Hang on, this makes people feel really bloody good’. And yeah, it feels heartfelt … it is validating, and is a beautiful thing to do” (11). Most of the *Wellness Warriors* identified that the exercise provided them with a way to appreciate one another and challenged them to see unique qualities in their colleagues that they had never seen before. This changed how they interacted with one another in the workplace. For example, a *Wellness Warrior* who would never visit the manager’s office before explained: “ … now I feel confident to walk in there and he’ll sit there and listen … I feel that the training gave me the confidence to do that … ” (06).

### Thinking and feeling differently about work

Through their experiences of the *Wellness Warrior* training and focusing on their own wellbeing, *Wellness Warriors* described that they started to think and feel differently about work including being more creative at work, changing workplace relations and having permission to slow down.

#### Bringing creativity to work

One aspect of thinking and feeling differently about work was “bringing creativity to work”. This was achieved through the creativity in the training which allowed *Wellness Warriors* to think differently about their role in the workplace. *Wellness Warriors* identified that to them creativity was just about being different and doing something they would not usually have done. For example:

The fact is now, the aftermath of the fires created this wellness training, the SEED Program, coffee buddies, quiet room, all these wonderful and creative tools. Now we have coping strategies that we can direct our colleagues to when they’re struggling. I believe those strategies and tools also let us have a bit of fun which we didn’t have before and that in itself connects us (06).

The *Wellness Warrior* training thus inspired creative spaces and tools to permeate the workplace.

#### Changing workplace relationships

Many of the *Wellness Warriors* shared that the *Wellness Warrior* training improved their relationships at work by providing them with strategies to identify the importance of interpersonal skills such as smiling or joking with their colleagues:

I feel more connection to the team and the fact that I can just walk through the ward and show a smile, have a joke, just help with fostering that positive atmosphere and energy in the place … it’s not just all about clinical care, and it’s not just about us healthcare professionals existing independently in a work environment, the training [*Wellness Warrior* training] showed us that we are in fact all connected and we can do it [work] in a better way (03).

Placing more emphasis on workplace relationships resulted in a more positive atmosphere including better connection with the management. Many of the *Wellness Warriors* felt that the bushfires were the catalyst for the hospital and the broader healthcare organization to think and feel differently about work. As one of the *Wellness Warriors* explained:

I think we were shown how valuable wellness actually is, we’ve had bushfires thrown at us and we’ve had floods and now we’ve got virus and yet this [*Wellness Warrior* training] has been able to sustain the people through the entire way and I don’t think we can underestimate that (07).

Consequently, several *Wellness Warriors* discussed that participating in the *Wellness Warrior* training allowed them to recognize the importance of investing in themselves, in order to have the ability to support the wellbeing of others in the workplace.

#### Having permission to slow down

*Wellness Warriors* discussed how the presence of the workplace wellness intervention gave them permission to slow down at work. This was unusual given the normally fast pace in healthcare systems. Many of the *Wellness Warriors* identified that they felt supported by their workplace to think differently about their own wellbeing, which had a flow on effect to others. They described that it was okay not to address everything at once and just slow down. One *Wellness Warrior* reflected on her past experiences of nursing when she claimed there was more time to slow down, stressing that the *Wellness Warrior* training gave her permission to return to a similar practice:

… in nursing, you used to be able to sit, you had the time … but things have changed. We can be technicians when it comes to all the machinery and stuff like that, but humans don’t change, they haven’t changed, so why should nursing and medicine change in that way? … it’s [*Wellness Warrior* training] given me more pause to listen to people again and be more patient … I think the intention for helping someone has always been the same, but how to do it … it’s [*Wellness Warrior* training] a framework (02).

The training thus provided a platform for *Wellness Warriors* to learn new skills and strategies to think and feel differently about work. They highlighted that bringing creativity to work, changing workplace relationships, and having permission to slow down helped sustain their wellbeing in the aftermath of the bushfires and provided them with the ability to support others.

### Restoring faith in healthcare leadership

Many of the *Wellness Warriors* believed that leadership support is essential to implementing and sustaining workplace wellness initiatives like SEED and the *Wellness Warrior* training. Most *Wellness Warriors* reflected that the *Wellness Warrior* training restored their faith in healthcare leadership as their leaders showed humility and modelled vulnerability. *Wellness Warriors* thus hoped to sustain the workplace wellness experiences for themselves and introduce it to others.

I’m pleased that management have been so supportive of this because it is innovative. It makes us feel as a workforce or as one of the people who was invested time and money to train, it makes us feel valued. It makes us actually want to give our workplace more because it inspires us (05).

The *Wellness Warrior* training allowed *Wellness Warriors* to feel valued and recognized by their leaders. Many *Wellness Warriors* stated that they felt more confident to approach and interact with their managers following the training:

Before the training I had never been to my managers office, in the three years I had worked here. Now I feel confident to walk in there and he will sit there and listen. Listen to something that might be trivial to him but was actually quite powerful for my patients and their family. I felt that after doing the training I gained confidence to do that, because I have never done that, ever, ever (04).

Several *Wellness Warriors* also discussed how participating in the training allowed them to recognize their own leadership qualities and enhanced their interaction with others. This was particularly useful for the *Wellness Warriors* who were in leadership roles themselves: “The skills from the *Wellness Warriors* in isolation have probably enhanced my COVID response and staff management response, adding a layer of listening, consideration and humanity” (08).

#### Leaders showing humility and modelling vulnerability

A turning point in restoring *Wellness Warriors* faith in healthcare leadership, was, in the words of one of the *Wellness Warrior* trainees: “leaders showing humility and modelling vulnerability” (06). *Wellness Warriors* emphasized that their leaders were honest and transparent immediately after the bushfires, admitting that they did not have the answers and were open to shared decision-making with the frontline staff. *Wellness Warriors* also discussed the importance of their leaders visiting the hospital site and showing care and commitment to staff wellbeing and recovery. A particular *Wellness Warrior* shared her experience of listening to a leader recount the lessons she had learnt when working in her managerial role during and in the aftermath of another natural disaster:

It showed that she [leader] cared, it showed that she’s learnt from her previous running of the other catastrophes to ours and I think we can only get better. I think the outcome of it all is that she [leader] realized what didn’t work previously and made changes for our community and district … I’m really grateful to her for doing that (07).

This was a powerful experience for *Wellness Warriors* as they were shown the openness, transparency, and vulnerability of their leaders in a disaster environment, acknowledging the need to put people before process. A consequence of this was that there was a levelling of the playing field where hospital staff were treated as an equal. Other *Wellness Warriors* also expressed having never encountered such a sense of support from their leaders in their time working in healthcare. *Wellness Warriors* believed that the openness of leaders to support wellness in the workplace was a result of the training:

Management have been great to get us here and even today, it is busy up there and we are actually working a way of doing it [supporting each other]. It has taken a lot of shuffling, but we have never seen management this open to new ways of working. Management is behind this, management stepped out of their offices and had to get back on the floor again for a little while so that we can do the training (02).

#### Wanting to sustain this experience

All *Wellness Warriors* hoped to sustain their experiences by continuing to invest in themselves and others as *Wellness Warriors*. Most *Wellness Warriors* stated that the *Wellness Warrior* training restored their faith in healthcare leadership, advocating for all healthcare workers to have an experience of participating in the training:

My dreams are that this [*Wellness Warrior* training] is taught in all the orientation packages …, would be a module taught in nursing at the university … that our management attend it … and that it’s something that’s not just taught to our district but taught to all nurses, all colleagues, all allied health colleagues, everybody in that industry (15).

All *Wellness Warriors* talked about wanting to sustain this experience by encouraging others to embed wellness into the everyday practices of the workplace. Many of the *Wellness Warriors* expressed that they feel as though they are the pioneers of wellness in healthcare and hope for the training programme not to fade away. They identified that for this experience to be sustained, they need to actively support one another as *Wellness Warriors* as well as receive ongoing support from healthcare leadership.

## Discussion

Communities experience countless challenges in the aftermath of disasters, including the pressures and double burden placed on healthcare workers who are often personally affected by the disaster and still expected to continue providing care to their patients. *Wellness Warriors* exemplify how a healthcare organization responded to the impact of natural disasters on their staff. Participants in this study described new ways of connecting with colleagues and responding to workplace stress following their *Wellness Warrior* training. The training aimed to challenge their previous ways of working in teams and enhance resilience and recovery processes post disaster. It built on a strengths-based approach to ascertain the needs of healthcare staff, ensuring that the training was based on the principle of “for the staff by the staff”.

The personal impact of the *Wellness Warrior* training relates to new skills and strategies staff developed during and after their participation. All *Wellness Warriors* articulated that participating in the training provided transformation, healing, and growth, as they had the opportunity to pause to listen and connect with their colleagues. Learning how to pause to listen was also described as a revelation for many, seeing their roles in conversations and relationships differently. This finding is supported by existing literature, stating that psychosocial training in listening skills following disasters has the potential to result in post-traumatic growth (PTG) in healthcare staff, as they gain confidence and self-esteem about their role in the workplace (Brooks et al., 2019). Therefore, future post-disaster interventions could place a stronger emphasis on PTG as opposed to post-traumatic stress. Whilst not minimizing the challenges, post-disaster supports should be drawing on a community’s strengths and opportunities for growth (Harms et al., 2018).

Underpinned by a strengths-based approach, the *Wellness Warrior* training focused on engagement among healthcare staff through participation in activities with an emphasis on building interpersonal skills. With the exception of the Resilience and Coping for the Healthcare Community (RCHC) intervention post Hurricane Sandy, which focused on both individual and collective coping skills through a combination of groupwork and psychoeducation (Yuma et al., 2019), there is currently limited evidence regarding wellness interventions for healthcare staff in the immediate aftermath of a natural disaster. Consistent with the practices of the RCHC intervention, *Wellness Warriors* described that participating in the training allowed them to recognize the importance of having time to connect with their colleagues. All 18 *Wellness Warriors* highlighted that undertaking the training with their colleagues and learning new interpersonal skills had a significant impact on themselves and the people that are closest to them. This experience is also supported by Umeda et al. (2020) who recommend that the creation of peer support systems in healthcare can build a supportive and resilient team. Our findings thus suggest that focusing on interpersonal skills such as deep listening and learning how to build meaningful connections with others could support the implementation of future workplace wellness intervention responding to disasters and cries.

The Wellness Warrior training appeared to have changed the way the participating staff thought and felt about the workplace. Many *Wellness Warriors* reflected on the bushfires as a catalyst for transformation and restoration in the workplace. They emphasized that it gave them the permission to prioritize their own wellness and the wellness of others in the work, building collective care and resilience. Brooks et al. (2019) support this concept, by stating that building positive workplace relationships in times of disaster will in turn foster a “supportive workplace atmosphere” (p.5). Thus, training such as *Wellness Warriors* have the potential to transform the workplace culture, changing the way healthcare workers relate to the hospital as a service and to each other.

The leadership investment by managers and the executive team to support *Wellness Warriors* during and after the training was described as restoring their faith in healthcare leadership. In contrast to Filmer and Ranse (2013) who suggest that leaders should employ a military style approach to leadership to make time-critical decisions during a disaster, *Wellness Warriors* reported that both the district leaders and hospital management employed collaborative ways of working in times of crisis and emphasized the collective wellbeing of healthcare professionals. Consistent with these findings, Waugh and Streib (2006) and Dietchman (2013) emphasize the need for leaders to move away from a top-down and hierarchical model of leadership to more dynamic models that facilitate staff support and collaboration. Several *Wellness Warriors* described that a particular leader demonstrated aspects of dynamic leadership through sharing her personal experiences of working in another natural disaster. *Wellness Warriors* recalled this as a turning point for restoring their faith in healthcare leadership, as they were shown the value of leaders demonstrating humility and modelling vulnerability.

We believe this study can offer several implications for healthcare practice, policy, and research. Healthcare systems and organizations need to dedicate time and funding to implementing post-disaster supports for staff that ensure recovery and resilience and to generally promote mental health and wellbeing in the workplace. Specifically, healthcare leaders should consider promoting the development of tailored wellness interventions in the aftermath of disasters and beyond. As demonstrated by this study, strengths-oriented wellness programmes can have an immediate personal impact on healthcare staff and promote building more meaningful connections with their colleagues and leaders. In addition, the findings also suggest that strengths-based training can improve the overall culture of the hospital as a service, prompting healthcare staff to think and feel differently about the work. Leadership investment in workplace wellness, particularly following a disaster can thus improve staff’s trust in the broader healthcare system and restore their faith in leaders. Finally, more research is needed to identify and describe current workplace wellness interventions in healthcare settings and design, implement and evaluate new interventions.

### Strengths and limitations

The study had a relatively small sample from one regional area. In addition, the study focused on only one aspect of the broader wellness programme (SEED), therefore *Wellness Warrior’s* experiences may not necessarily be representative of all healthcare staff involved in SEED. Finally, due to the COVID-19 restrictions, interviews had to be conducted via telephone that created some challenges in building rapport and trust with staff.

Nevertheless, a strength of this study is that all 18 healthcare staff trained as *Wellness Warriors* participated in data collection, which ensured that the experiences and perspectives of all trainees were captured. Further, this study provides a localized perspective of the implementation of a training to support the wellbeing of healthcare staff immediately after a natural disaster demonstrating the importance of building on local knowledge to offer local solutions in times of disaster.

## Conclusion

Supporting the wellbeing of healthcare staff is paramount if they are to continue to practice effectively during and in the aftermath of disasters and the workplace has a responsibility to address staff needs. The study demonstrated that the *Wellness Warrior* training offered in the rural hospital provided healthcare staff with necessary knowledge and skills to respond to the mental health and recovery needs of their colleagues and their own in the aftermath of the bushfires and floods. *Wellness Warriors* learned how pause to listen, share stories, and connect with others. Ultimately, this enabled them to think and feel differently about their workplace, restoring their faith in healthcare leadership and the healthcare system in general. This transformational and restorative impact the wellness intervention had on staff was particularly important when they were faced with yet another crisis—the COVID-19 pandemic. Thus, intentional workplace efforts to take care of staff’s wellbeing needs not only ensure post-disaster recovery but also change the workplace culture and build their skills and resilience for handling inevitable future crises.

## Supplementary Material

Supplemental Material
