# Supplementary material for: Wellness Warriors: a qualitative exploration of healthcare staff learning to support their colleagues in the aftermath of the Australian bushfires
Source: Int J Qual Stud Health Well-being. 2023 Jan 19;18(1):2167298. doi: 10.1080/17482631.2023.2167298 (PMC9858529; doi:10.1080/17482631.2023.2167298)
Supplement: Supplemental Material [file ZQHW_A_2167298_SM0073.docx]

**Supplemental Material**

**Wellness Warriors Interview Guide**

**Demographic Questions**

1. What is your role at Milton Hospital?
2. How long have you been in your current role?
3. How long have you worked in Milton Hospital? (Or in the Illawarra Shoalhaven Local Health District):
4. How long have you lived in the local area?

**Interview Questions**

1. Why did you get involved in the Wellness Warrior training?
2. What did you learn during the Wellness Warrior training?
   1. Is there a lesson or moment in the training that you particularly remember?
3. Have you used the skills you learnt in the Wellness Warrior training at work?
   1. Can you think of some stories that will show me this?
4. Do you think that being a Wellness Warrior helped you support others (e.g., colleagues, carers or family) during a challenging time?
   1. Can you tell me a story of a particular moment when you noticed this?
5. What kind of ongoing support do you need to be a Wellness Warrior?
6. How could the Wellness Warrior training or role be improved in Milton Hospital?
7. What are your Wellness Warrior dreams?
8. Is there anything else you would like to say about the facilitators/management?
